# Patterns of glioblastoma treatment and survival over a 16-years period: pooled data from the German Cancer Registries

**DOI:** 10.1007/s00432-021-03596-5

**Published:** 2021-03-20

**Authors:** Ljupcho Efremov, Semaw Ferede Abera, Ahmed Bedir, Dirk Vordermark, Daniel Medenwald

**Affiliations:** 1 Institute for Medical Epidemiology, Biometrics and Informatics (IMEBI), Interdisciplinary Center for Health Sciences, Medical School of the Martin-Luther-University Halle-Wittenberg, Magdeburger Str. 8, 06112 Halle (Saale), Germany; 2 Department of Radiation Oncology, Martin-Luther-University, Halle (Saale), Germany

**Keywords:** Glioblastoma, Temozolomide, Overall survival, Cancer registry

## Abstract

**Introduction:**

Glioblastoma multiforme (GBM) is a primary malignant brain tumour characterized by a very low long-term survival. The aim of this study was to analyse the distribution of treatment modalities and their effect on survival for GBM cases diagnosed in Germany between 1999 and 2014.

**Methods:**

Cases were pooled from the German Cancer Registries with International Classification of Diseases for Oncology, third edition (ICD-O-3) codes for GBM or giant-cell GBM. Three periods, first (January 1999–December 2005), second (January 2006–December 2010) and a third period (January 2011–December 2014) were defined. Kaplan–Meier plots with long-rank test compared median overall survival (OS) between groups. Survival differences were assessed with Cox proportional-hazards models adjusted for available confounders.

**Results:**

In total, 40,138 adult GBM cases were analysed, with a mean age at diagnosis 64.0 ± 12.4 years. GBM was more common in men (57.3%). The median OS was 10.0 (95% CI 9.0–10.0) months. There was an increase in 2-year survival, from 16.6% in the first to 19.3% in the third period. When stratified by age group, period and treatment modalities, there was an improved median OS after 2005 due to treatment advancements. Younger age, female sex, surgical resection, use of radiotherapy and chemotherapy, were independent factors associated with better survival.

**Conclusion:**

The inclusion of temozolomide chemotherapy has considerably improved median OS in the older age groups but had a lesser effect in the younger age group of cases. The analysis showed survival improvements for each treatment option over time.

**Supplementary Information:**

The online version contains supplementary material available at 10.1007/s00432-021-03596-5.

## Introduction

Glioblastoma, also known as glioblastoma multiforme (GBM), is the most common primary malignant brain tumour characterized by an infiltrative growth pattern and a low survival rate, with only 6.8% of patients surviving five years post-diagnosis according to the 2019 Central Brain Tumour Registry of the United States (CBTRUS) Statistical Report (Ostrom et al. 2019). The reported incidence rate worldwide varies between 0.59 (South Korea) (Lee et al. 2010), 3.19 (USA) (Thakkar et al. 2014), 3.3 (France) (Fabbro-Peray et al. 2019), and 3.8 (Sweden) (Eriksson et al. 2019) per 100,000 persons.

The ‘standard of care’ treatment for GBM is maximal safe surgical resection, followed by radiotherapy with concurrent and adjuvant temozolomide (TMZ) chemotherapy (Al-Holou et al. 2020). The inclusion of TMZ chemotherapy in treatment guidelines was based on the results from a clinical trial by (Stupp et al. 2005). This landmark trial demonstrated that the addition of concomitant and adjuvant TMZ to radiotherapy provided additional survival benefit to patients, with an increase in median overall survival (OS) of 14.6 months compared to 12.1 months in the radiation only arm. The addition of TMZ is considered a major contributor to survival improvement, from the median 6.9 months in those diagnosed during 1995–1996 to 10.3 months in the period 2010–2015, with 2-year survival peaking at 18%, shown by a Swedish single centre study (Eriksson et al. 2019). Several studies from the US compared survival rates in the periods pre- and post-TMZ approval, predominantly based on the Surveillance, Epidemiology and End Results (SEER) Program database (Zhu et al. 2017; Johnson and O'Neill 2012; Darefsky et al. 2012), but also from the Veterans Health Administration (VHA) dataset (Dubrow et al. 2013). These studies confirmed that survival improvement for GBM patients overlapped with the addition of TMZ to standard therapy protocols. Several European studies reported similar findings (Fabbro-Peray et al. 2019; Eriksson et al. 2019; Scoccianti et al. 2010; Ronning et al. 2012; Hansen et al. 2018). However, even with high compliance to therapy regimens, cancer registry-based studies usually report shorter median OS than those shown by clinical trials.

The aim of this study was to evaluate the distribution of treatment modalities after GBM diagnosis, such as surgery, radiotherapy, and chemotherapy, encompassing 16 years (1999–2014) of data collection by the German Cancer Registries, and to analyse their effect on survival. We hypothesized that there would be an improvement in survival due to technological advancements and more effective treatment options for GBM patients between the first and last period.

## Methods

### Study population

Data were provided by ‘The Centre for Cancer Registry Data’ at the Robert Koch Institute (RKI) (Kraywinkel et al. 2014) that is tasked with annually merging cancer data from 16 German federal states’ individual registries. This data can be freely requested for research purposes. The dataset contained the following variables: date of birth, date of diagnosis, date of death, sex, histology, grading, tumour localization, International Statistical Classification of Diseases (ICD-10), International Classification of Diseases for Oncology, third edition (ICD-O-3) codes, federal-state residence and treatment modalities. Details on administered radiation doses or specific chemotherapy regimens were not available. We analysed only cases (*n* = 44,865) with ICD-O-3 codes 9440 (glioblastoma, 98.9%) or 9441 (giant-cell glioblastoma, 1.1%), and excluded patients with other codes (GBM dataset).

Data quality was assessed by checking the proportion of death certificate only (DCO) or autopsy only cases. The recommendation from The European Cancer Registry-based Study on Survival and Care of Cancer Patients (EUROCARE-5 Study) is that registries should have below 13% DCO cases (Rossi et al. 2015). In the GBM dataset, the proportion of DCO cases was 10.8%. The accuracy of the stated diagnosis is likely to be higher if it is based on histological examination (Bray and Parkin 2009). In the current dataset, diagnosis of GBM was histologically verified in 85.7% of cases. Furthermore, the dataset had no missing information on variables deemed important for the assessment of quality and completeness of registry data, such as age at diagnosis, sex and information on federal-state residence (Bray and Parkin 2009).

Cases were excluded if they were diagnosed by means of an autopsy or DCO (*n* = 4364), less than 18 years old (*n* = 358) or had missing information on survival status and exact date of death (*n* = 5). For cases that were recorded as deceased, but had missing exact date of death (*n* = 1003), a 0.5 months follow-up time was added, assuming that they survived at least 15 days after diagnosis. The final analysed sample contained 40,138 cases.

### Definition of periods and study outcome

Cases were divided into three groups according to the year of diagnosis. We defined the period between January 1999 and December 2005 as the pre-TMZ chemotherapy era, January 2006–December 2010 as the period when TMZ chemotherapy became part of the standard therapy protocol (TMZ era), and the period between January 2011–December 2014 as the modern era. Cases were censored at 31st December 2014 (last possible date to be included in the GBM dataset) or after 30 months of follow-up, which ever came first.

The main study outcome was median OS. First, the median OS for all cases was calculated and then evaluated separately for the three defined time periods. Secondly, median OS of cases who received surgical treatment and radiotherapy was compared to patients who received all three treatment modalities, following similar categorization from two previous major clinical trials (Stupp et al. 2005; Perry et al. 2017). Thirdly, age-stratified analysis of median OS was performed for each period. Finally, we analysed overall distribution of each treatment modality.

### Statistical analyses

Calculation of median OS was according to the Kaplan–Meier (KM) method. Survival probabilities were graphically presented with KM plots and subgroups were compared with the log-rank test. Cox proportional-hazards models were used to examine survival for each period by computing hazard ratios (HR) with 95% confidence intervals (CI). Models were adjusted for age, sex, and each treatment modality. Information on treatment was available as a dichotomous variable (surgery yes/no, radiotherapy yes/no, chemotherapy yes/no). The significance of three interaction terms (each treatment modality × time period) was also explored in the final model. No competing risk analysis was performed as GBM has characteristic short duration of survival and to avoid bias from erroneous recording of cause of death**.** Sensitivity analysis was performed to determine the stability of the HRs when federal states with more than 30% missing information on treatment modalities were excluded. Logistic regression models were used to determine if age, sex and time period were predictors for missing information on treatment. Additional information on the statistical analyses is included in the Online Supplemental Resource. All analyses were performed with SAS 9.4 (SAS Institute, Cary, NC, USA).

## Results

### Demographic characteristics

The GBM dataset contained a total of 40,138 adult cases, diagnosed between 1999 and 2014 (Table 1). During this period, 34,883 (86.9%) of patients died. The mean age at diagnosis was 64.0 ± 12.4 years (range 18–100), with a trend toward diagnosis at an older age for the third period. Most cases (60%) were between 60 and 79 years of age. A slight decrease in number of patients was observed for the age categories 18–49 and 60–69 years, while an increase was seen in the 70–79 and ≥ 80 age categories, when comparing the first and third period. Overall, the majority of patients were men (*n* = 23,003, 57.3%), with the sex ratio similar in all three periods.

**Table 1:** Demographic and clinical characteristics of GBM cases

Characteristics	Total (*n* = 40,138)	Period 1999–2005 (*n* = 10,932, 27.2%)	Period 2006–2010 (*n* = 14,836, 37%)	Period 2011–2014 (*n* = 14,370, 35.8%)
Age, years ± SD	64.0 ± 12.4	62.4 ± 12.3	64.1 ± 12.3	65.1 ± 12.4
Age categories				
18–49, *n* (%)	5455 (13.6)	1739 (15.9)	1995 (13.4)	1721 (12.0)
50–59, *n* (%)	7867 (19.6)	2155 (19.7)	2899 (19.5)	2813 (19.6)
60–69, *n* (%)	12,396 (30.9)	3922 (35.9)	4607 (31.1)	3867 (26.9)
70–79, *n* (%)	11,494 (28.6)	2587 (23.7)	4250 (28.6)	4657 (32.4)
≥ 80, *n* (%)	2926 (7.3)	529 (4.8)	1085 (7.3)	1312 (9.1)
Women, *n* (%)	17,135 (42.7)	4694 (42.9)	6322 (42.6)	6119 (42.6)
Men, *n* (%)	23,003 (57.3)	6238 (57.1)	8514 (57.4)	8251 (57.4)
Median follow-up time, months (IQR)	9.0 (4.0–18.0)	9.0 (4.0–18.0)	10.0 (4.0–21.0)	8.0 (4.0–16.0)
Survival				
Median OS, months (95% CI)	10.0 (9.0–10.0)	9.0 (9.0–9.0)	10.0 (10.0–10.0)	10.0 (10.0–10.0)
2-year survival, % (95% CI)	19.1 (18.7–19.5)	16.6 (15.9–17.3)	20.6 (20.0–21.3)	19.3 (18.6–20.1)
Treatment				
Surgery, yes *n* (%)	21,560/26,215^a^ (82.2)	5703 (74.0)	8054 (84.1)	7803 (87.3)
Radiotherapy, yes *n* (%)	18,941/25,339^a^ (74.7)	5381 (71.9)	6748 (75.5)	6812 (76.4)
Chemotherapy, yes *n* (%)	11,075/22,728^a^ (48.7)	1909 (28.3)	4606 (54.9)	4560 (60.0)
Immunotherapy, yes *n* (%)	88/13,463^a^ (0.6)	20 (0.4)	25 (0.5)	43 (1.1)

OS – overall survival, SD – standard deviation, IQR – interquartile range, CI – confidence interval

^a^The first number is the cases that got the treatment modality (coded as yes), while the second number is the total number of cases with non-missing data for this variable

### Treatment patterns

Surgery and radiotherapy were the most common treatment modalities with 82.2% and 74.7%, respectively (Table 1). There was an increase in the use of chemotherapy over time, from 28.3% in the first period to 54.9% in the interim and 60.0% in the third period. Among the cases in the dataset, the highest proportion with the absence of cancer-related therapy were in the age group 70–79 years (36.3%), with only 1.6% of those above 80 years receiving multimodal therapy. Use of multimodal therapy was more common for men and younger age groups. As expected, median follow-up was shortest in the absence of cancer-related treatment group (3 months) compared to the multimodal treatment group, which had the longest follow-up time (14 months) (Table 2).

**Table 2:** Distribution of treatment modalities

	Absence of cancer-related treatment	Surgery only	Surgery and Radiotherapy	Surgery, radiotherapy, chemotherapy
No. of cases	2052	3822	4145	9038
Median follow-up, months (IQR)	3.0 (1.0–9.0)	6.0 (2.0–14.0)	9.0 (4.0–15.0)	14.0 (8.0–23.0)
Age, years ± SD	69.4 ± 12.4	65.3 ± 12.3	65.4 ± 11.4	60.1 ± 11.7
Age categories				
18–49, *n* (%)	153 (7.4)	443 (11.6)	442 (10.7)	1668 (18.5)
50–59, *n* (%)	238 (11.6)	662 (17.3)	697 (16.8)	2396 (26.5)
60–69, *n* (%)	517 (25.2)	1149 (30.1)	1311 (31.6)	3059 (33.9)
70–79, *n* (%)	744 (36.3)	1247 (32.6)	1434 (34.6)	1768 (19.5)
80 + , *n* (%)	400 (19.5)	321 (8.4)	261 (6.3)	147 (1.6)
Women, *n* (%)	944 (46.0)	1685 (44.1)	1813 (43.7)	3636 (40.2)
Men, *n* (%)	1108 (54.0)	2137 (55.9)	2332 (56.3)	5402 (59.8)

*SD* standard deviation, *IQR* interquartile range

### Median OS

**Fig. 1:**
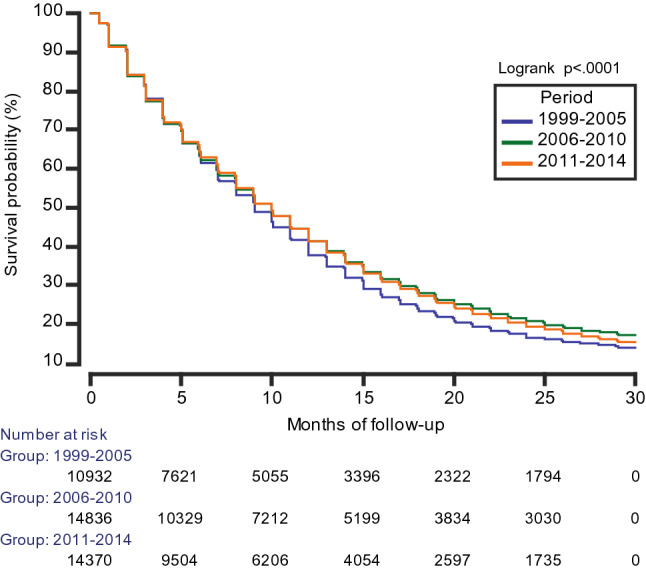
Kaplan–Meier plot comparing the survival function in the three-time periods

**Fig. 2:**
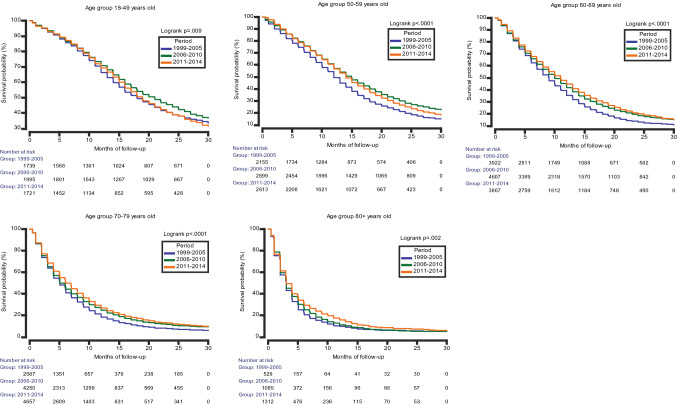
Kaplan–Meier plots comparing the survival function between periods stratified by age at GBM diagnosis

The median OS was 10.0 (95% CI 9.0–10.0) months (Table 1). The 2-year OS was 19.1%, with a slight increase over time, from 16.6% in the first period, to 20.6% and 19.3% in subsequent periods. Figure 1 shows KM plots of OS between the three periods. The difference in survival between the first and second period, and between the first and third period were significant (log-rank test, *p* < 0.0001), while the survival curves for the second and third period were nearly identical (log-rank test, *p* = 0.04). Table 3 shows the different median OS between cases treated with surgery and radiotherapy, and with all three treatment modalities, stratified by period and by age groups. While the median OS became worse with advancing age, an improvement in survival after 2005 for the older age groups (70–79 and above 80-years) was observed, especially for those that also received chemotherapy. There was a decrease in median OS on multimodal therapy for the 18–49 age group in the third period (2011–2014). Figure 2 displays KM plots comparing age-stratified median OS for each period. The lower survival for the younger age groups (18–49, 50–59 years), when comparing between the second and third period, was most noticeable after approximately 15 months.

**Table 3:** Median OS stratified by age group, period and treatment modality

Age group	Surgery and radiotherapy			Surgery, radiotherapy and chemotherapy		
	Median OS, months (95% CI)			Median OS, months (95% CI)		
	1999–2005	2006–2010	2011–2014	1999–2005	2006–2010	2011–2014
18–49	19.0 (18.0–21.0)	23.0 (22.0–25.0)	21.0 (19.0–22.0)	21.0 (18.0–23.0)	24.0 (22.0–26.0)	20.0 (19.0–22.0)
50–59	13.0 (13.0–14.0)	17.0 (16.0–18.0)	16.0 (15.0–17.0)	14.0 (13.0–16.0)	17.0 (16.0–18.0)	16.0 (15.0–17.0)
60–69	11.0 (10.0–11.0)	13.0 (12.0–13.0)	14.0 (13.0–14.0)	13.0 (12.0–13.0)	14.0 (13.0–14.0)	14.0 (13.0–15.0)
70–79	7.0 (7.0–8.0)	8.0 (8.0–9.0)	9.0 (9.0–9.0)	10.0 (8.0–12.0)	10.0 (9.0–11.0)	11.0 (10.0–11.0)
≥ 80	5.0 (4.0–6.0)	6.0 (5.0–7.0)	6.0 (5.0–8.0)	6.0 (2.0–18.0)	7.0 (5.0–9.0)	7.0 (5.0–11.0)

*OS* overall survival, *CI* confidence interval

### Survival adjusted for confounders and treatment modalities

Cox proportional-hazards models revealed that younger age, female sex, surgical resection, use of radiotherapy and chemotherapy, were independent factors associated with better survival (Table 4). We observed a progressive increase in HRs from younger toward older age groups. The oldest age group (≥ 80 years) had the highest hazards ratio 4.36 (95% CI 4.05–4.70) after adjusting for all available confounders. We observed lower hazards for the second, 0.85 (95% CI 0.83–0.88), and third period, 0.83 (95% CI 0.80–0.85) (Model 1), taking the 1999–2005 period as reference. There was no survival difference between the first and third period after adjustment for chemotherapy (Model 3). However, there was a significant interaction (*p* < 0.0001) between each treatment modality and the defined periods, indicating a difference in treatment effect across time. The survival improvement over time is indicated by decreasing HRs from the first to the third period in an interaction analysis (Table 5). Use of chemotherapy versus no chemotherapy showed a decreasing HR from 0.75 (95% CI 0.70–0.80) in the 1999–2005 period to 0.66 (95% CI 0.61–0.70) in the 2011–2014 period. Similarly, there was improved survival with surgical resection, HR 0.86 (95% CI 0.81–0.91) in 1999–2005 to 0.69 (95% CI 0.64–0.74) in the 2011–2014 period. The improvement of survival over time with radiotherapy was more modest, with HR 0.91 (95% CI 0.86–0.97) for the first to 0.83 (0.77–0.89) for the third period, respectively.

**Table 4:** Cox proportional-hazards models

Variables	Model 1^a^	Model 2^a^	Model 3^a^
	HR (95% CI)	HR (95% CI)	HR (95% CI)
Age groups			
18–49	Reference	Reference	Reference
50–59	1.52 (1.45–1.58)	1.57 (1.49–1.66)	1.55 (1.47–1.64)
60–69	2.00 (1.92–2.08)	2.03 (1.93–2.13)	1.99 (1.89–2.10)
70–79	3.05 (2.93–3.17)	3.10 (2.95–3.26)	2.94 (2.79–3.10)
≥ 80	4.86 (4.62–5.12)	4.69 (4.37–5.03)	4.36 (4.05–4.70)
Men	Reference	Reference	Reference
Women	0.94 (0.92–0.96)	0.93 (0.90–0.95)	0.92 (0.89–0.95)
Period 1999–2005	Reference	Reference	Reference
Period 2006–2010	0.85 (0.83–0.87)	0.87 (0.84–0.91)	0.96 (0.92–0.99)
Period 2011–2014	0.82 (0.80–0.85)	0.86 (0.83–0.90)	1.00 (0.96–1.04)
Surgery, No	–	Reference	Reference
Surgery, Yes	–	0.70 (0.68–0.73)	0.74 (0.71–0.77)
Radiotherapy, No	–	Reference	Reference
Radiotherapy, Yes	–	0.72 (0.70–0.74)	0.87 (0.84–0.91)
Chemotherapy, No	–	–	Reference
Chemotherapy, Yes	–	–	0.70 (0.67–0.72)

^a^Model 1 is age and sex adjusted, Model 2 is Model 1 + surgery and radiotherapy, Model 3 is Model 2 + chemotherapy. Abbreviations: HR – hazard ratio; CI – confidence interval

**Table 5:** Interaction analysis of use of treatment vs no treatment for each period

Interaction analysis	Period 1999–2005 HR (95% CI)	Period 2006–2010 HR (95% CI)	Period 2011–2014 HR (95% CI)
Surgery, yes vs no	0.86 (0.81–0.91)	0.64 (0.60–0.68)	0.69 (0.64–0.74)
Radiotherapy, yes vs no	0.91 (0.86–0.97)	0.88 (0.83–0.94)	0.83 (0.77–0.89)
Chemotherapy, yes vs no	0.75 (0.70–0.80)	0.72 (0.68–0.76)	0.66 (0.61–0.70)

For the analysis, Cox Model 3 was used with the additional inclusion of all three interaction terms (treatment modalities × time period). Abbreviations: HR—hazard ratio, CI—confidence interval

### Sensitivity analysis and predictors for missing treatment information

To explore the effect of missing treatment information on the stability of HRs, we excluded data from federal states (*n* = 6) that had more than 30% missing information on treatment modalities. With a sample size of *n* = 23,055, we repeated the Cox proportional-hazards analysis (Online Supplemental Resource Table 1). The sensitivity analysis revealed that the HRs were relatively robust, except for a decrease in HRs observed in the oldest age group (Model 2 and 3) and the two latter time periods (Model 1). This is in line with the results from the logistic regression analysis (Online Supplemental Resource Table 2), where diagnosis at an older age and cases from the period 2006–2010 and 2011–2014 presented with higher odds for having missing information on treatment modalities.

## Discussion

Our results showed that advancements in treatment routines, especially after 2005, led to the improvement of survival based on the Cox model estimates and improved 2-year survival for GBM patients in Germany. First, the median OS was 10 months, which is in line with results from other cancer registry studies (Fabbro-Peray et al. 2019; Eriksson et al. 2019; Zhu et al. 2017; Johnson and O'Neill 2012; Hansen et al. 2018) and the clinical trial in elderly patients by Perry et al. (Perry et al. 2017). Analyzing the age distribution, cases were mostly in the older age categories, which could explain the lower OS in the present study compared to the trial by Stupp et al. (2005), which had an upper limit (71 years) on age when enrolling participants. Taking into account that clinical trials perform a rigorous selection of patients, with a different distribution of comorbid conditions and better access to treatment, median OS is usually higher than what is observed in population-level studies (Pulte et al. 2019). Secondly, we observed that the median OS, when stratified by age, treatment and time period, improved after 2005. The addition of TMZ chemotherapy to surgical resection and radiotherapy increased median OS for the older age groups, however, it had no to little effect in the younger age groups. The interaction analysis revealed that the effect of treatment modalities changed over time, with the biggest improvement stemming from chemotherapy use and advancements in surgical treatment. We cannot answer based on currently available data whether this is a result of improved precision or the extent of surgery. However, regarding chemotherapy, the only chemotherapy agent approved for first-line treatment of GBM has been TMZ, to which we can attribute most of the improvement. Finally, the absence of cancer-related treatment was more common in the older age groups, while multimodal therapy was more customary in the younger group.

A key factor that influenced survival was the age at diagnosis. The survival disparity between the different age groups was expected since survival declines with increasing age (Laperriere et al. 2013). A recent clinical trial by Perry et al. examined the treatment paradigm for patients older than 65 years, and concluded that the addition of TMZ to short-course radiotherapy resulted in longer survival than radiotherapy alone (Perry et al. 2017). This is relevant for clinical practice, taking into account that 35% of our cases were in the 70 and older age category. Additionally, molecular prognostic factors for GBM are correlated with age. These include O^6^-methylguanine-DNA methyltransferase (MGMT) gene promotor methylation and isocitrate dehydrogenase (IDH1) mutations (Weller et al. 2009). About 10% of GBMs are IDH gene mutated and this is associated with a better prognosis, while typically GBMs are IDH wild-type. Elderly patients are more likely to have IDH wild-type, which indicates a shorter survival compared to younger patients (Zhu et al. 2017). Additionally, older patients are burdened with more comorbidities and have more treatment-related side effects (Brandes et al. 2009). However, this group often has methylation of the MGMT promotor, which offers chemosensitivity and increased benefit from TMZ treatment (Perry et al. 2017; Wick et al. 2012; Hegi et al. 2005).

Survival rates for patients with GBM have shown little notable improvement in the last two decades. Our results are comparable to the median OS and 2-year survival rates reported by other population-based registry studies using SEER, VHA, CRN (Cancer Registry of Norway) (Ronning et al. 2012), the FBTDB (French Brain Tumor Database) (Fabbro-Peray et al. 2019) and the Danish Neuro-Oncology Registry (Hansen et al. 2018). According to the study by Dubrow et al. (2013), addition of TMZ chemotherapy to standard therapy protocols explained the improved survival observed on a population level, even though they showed much lower median OS (6.4 months). Our results are comparable to the study by Zhu et al. (2017) that reported 10 months median OS and a 2-year survival rate of 16.8%. Other European studies, such as from France, reported 11.2 months (Fabbro-Peray et al. 2019), Norway, 8.3 and 10.1 months in the pre-TMZ and TMZ eras, respectively (Ronning et al. 2012), and Denmark, 11.2 months (Hansen et al. 2018). A clear explanation why there is little change in survival from GBM in Germany during 16 years is difficult. One possible reason could be that cases diagnosed at an older age, whose numbers increased substantially due to demographic changes or improved case ascertainment in the cancer registry, are subjected to less aggressive treatment protocols. A study from Fabbro-Perray et al. (2019), stated that in ‘real-world’ setting, only 60% of patients initiate standard treatment, and while we are unable to confirm this from our data, the overall use of surgery, radio- and chemotherapy was relatively high and increased in subsequent periods, meaning this could not completely explain the discrepancy.

Glioblastoma multiforme is a costly disease due to hospitalization and life-years lost in the post-diagnosis period, hospice care, and claims for physical, occupational and speech therapy (Aly et al. 2019). Before our analysis, we expected a more substantial improvement in survival of GBM patients, considering advances in surgical techniques and the introduction of postoperative radio-chemotherapy with adjuvant chemotherapy as a standard treatment after 2005. The implementation of neuronavigation and the introduction of intraoperative 5-aminolevulinic acid (5-ALA) fluorescent microscopy have enhanced the possibility to achieve a better gross total tumor resection. Our analyses revealed that surgery is associated with better survival, as also reported by another study (Al-Holou et al. 2020). The effect of radiotherapy advancements was more modest even though there were several novel radiotherapy choices. Intensity-modulated radiation therapy represents an advance option in terms of conformal treatment. Additionally, tumor-treating fields (TTFs) are an important cancer treatment modality with antimitotic effects against rapidly dividing tumor cells (Fabian et al. 2019). This is an upcoming new standard of care for both newly diagnosed and recurrent GBM cases (Kazda et al. 2018). More aggressive treatment of recurrent disease, with the application of stereotactic radiotherapy and improvements in palliative care, might also contribute to better survival. Regarding chemotherapy, the use of TMZ has been consistently associated with improved survival. Co-administration of radiotherapy and TMZ has nearly tripled the 2-year survival of GBM patients in the last two decades from 10 to 27% and quadrupled to 47% in patients with MGMT promotor methylation (Kazda et al. 2018). However, current clinical trials have reported improved chemotherapy benefits only in patients with prognostically positive molecular profiles, and this is important evidence toward the implementation of a personalized treatment strategy (Herrlinger et al. 2019; Bent et al. 2019). Several known and newly discovered chemotherapy and immunotherapy agents have been tested, offering modest or no improvement in median OS (Gilbert et al. 2014; Weller et al. 2017), but no second-line treatment has been established. Regardless of modern state-of-the-art treatment, due to the highly invasive nature of GBM, median OS remains between 10 and 14 months (Seyfried et al. 2019). Additional research is needed to improve the poor prognosis of these patients.

### Strengths and limitations

The strength of our study is that it uses a large, nationally representative database of German GBM patients, classified by the ICD-O-3 coding system, with satisfactory data quality and completeness of the information. Registry-based studies are considered to reflect more accurately ‘real-world’ settings. There are also some potential limitations. Cancer registration in Germany was not nationwide before 2009, which could influence the lower number of cases seen in the first period. Additionally, epidemiological cancer registries do not routinely collect information on specific chemotherapy drugs or the applied regimen. It is possible that some cases did not receive the standard TMZ treatment, but a different chemotherapy medication, such as lomustine. In the pre-2005 era, German guidelines recommended the use of carmustine plus teniposide or nimustine plus teniposide based on results from two clinical trials (Weller et al. 2003). However, one study has shown that the most frequent chemotherapy modality after 2005 is TMZ with a median duration of 2.4 months (Aly et al. 2019). Another limitation is that no additional variables associated with survival were collected, such as performance status, the extent of surgery and presence of comorbidities (Eriksson et al. 2019). Due to lack of information on comorbidities we cannot exclude confounding by indication, which is a limitation of observational data, as opposed to randomized controlled trials. In addition, the sensitivity analysis revealed that recent periods had more missing information on treatment modalities and this was exacerbated by older age at diagnosis. Thus, the interpretation of the main effect estimates remains challenging. Finally, data on molecular factors were unavailable.

## Conclusion

While the median OS did not improve significantly during the three periods, there was a slight increase in 2-year survival and a notable survival improvement after 2005 for all treatment modalities. The addition of TMZ chemotherapy improved survival more evidently in the older age groups but had a lesser effect in the younger age groups. Survival improvement depends on several factors and accentuates the need for a personalized strategy in GBM treatment management.

## Supplementary Information

Below is the link to the electronic supplementary material.

Supplementary file1 (DOCX 38 KB)
