# Supplementary material for: Patterns of glioblastoma treatment and survival over a 16-years period: pooled data from the German Cancer Registries
Source: J Cancer Res Clin Oncol. 2021 Mar 20;147(11):3381–90. doi: 10.1007/s00432-021-03596-5 (PMC8484256; doi:10.1007/s00432-021-03596-5)
Supplement: Supplementary file 1 — Supplementary file1 (DOCX 38 KB) [file 432_2021_3596_MOESM1_ESM.docx]

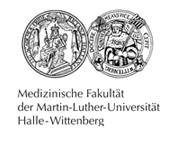
 (20/02/21 13:15)
Surv_glio_f_sup2_f.docx
*Journal of Cancer Research and Clinical Oncology*

# Patterns of glioblastoma treatment and survival over a 16-years period: pooled data from the German Cancer Registries

-Electronic supplementary material-

Short title: Glioblastoma treatment modalities and survival

**Correspondence to:**

Daniel Medenwald, MD, PhD

Institute for Medical Epidemiology, Biometrics and Informatics (IMEBI), Interdisciplinary Center for Health Sciences, Medical School of the Martin-Luther-University Halle-Wittenberg

Magdeburger Str. 8, 06112 Halle (Saale),

Germany

Email: Daniel.Medenwald@uk-halle.de

Ljupcho Efremov, MD, MSc^1,2^; Semaw Abera, MSc^2^; Ahmed Bedir, MSc^2^; Dirk Vordermark, MD, PhD^2^; Daniel Medenwald, MD, PhD^1,2^

**Affiliations**

^1^Institute for Medical Epidemiology, Biometrics and Informatics (IMEBI), Interdisciplinary Center for Health Sciences, Medical School of the Martin-Luther-University Halle-Wittenberg, Halle (Saale), Germany; ^2^Department of Radiation Oncology, Martin-Luther-University, Halle (Saale), Germany.

**Statistical analyses**

To comply with regulations on data anonymization, the cases in the GBM dataset have a fixed date, the 15 day of the month, for the dates of birth, diagnosis and death. Due to this, each case in the dataset contributes a minimum of 1 month to the survival analyses, except if cases were enrolled in the Registry at the last possible month (15^th^ December 2014) when they can contribute only half a month (study end-date 31^st^ December 2014) or they had status as deceased, but not exact date of death, in which case 0.5 months follow-up time was added, as a recommendation from the Robert-Koch Institute to take into account cases that died within the first couple of weeks. In order to make the interpretation of results easier, the median OS time in months has been rounded up to the nearest integer, if it was a fractional number.

**Online Resource Table 1**.

Sensitivity analysis Cox proportional-hazards models

| Variables | Model 1* | Model 2* | Model 3* |
| --- | --- | --- | --- |
|  | HR (95% CI) | HR (95% CI) | HR (95% CI) |
| Age groups |  |  |  |
| 18-49 | Reference | Reference | Reference |
| 50-59 | 1.49 (1.41-1.58) | 1.53 (1.44-1.62) | 1.51 (1.42-1.61) |
| 60-69 | 1.95 (1.85-2.06) | 1.96 (1.85-2.07) | 1.93 (1.82-2.05) |
| 70-79 | 2.95 (2.80-3.11) | 2.94 (2.77-3.12) | 2.78 (2.61-2.95) |
| ≥80 | 4.57 (4.26-4.90) | 4.30 (3.97-4.66) | 4.04 (3.71-4.40) |
| Men | Reference | Reference | Reference |
| Women | 0.94 (0.91-0.96) | 0.93 (0.90-0.96) | 0.93 (0.90-0.96) |
| Period 1999-2005 | Reference | Reference | Reference |
| Period 2006-2010 | 0.79 (0.76-0.81) | 0.85 (0.82-0.89) | 0.94 (0.90-0.98) |
| Period 2011-2014 | 0.72 (0.69-0.74) | 0.84 (0.81-0.88) | 1.00 (0.95-1.04) |
| Surgery, No | - | Reference | Reference |
| Surgery, Yes | - | 0.67 (0.65-0.70) | 0.71 (0.68-0.74) |
| Radiotherapy, No | - | Reference | Reference |
| Radiotherapy, Yes | - | 0.71 (0.68-0.73) | 0.85 (0.81-0.88) |
| Chemotherapy, No | - | - | Reference |
| Chemotherapy, Yes | - | - | 0.71 (0.68-0.74) |

The sensitivity analysis focussed only on data from federal states that reported less than 30% missing

information on treatment modalities. * Model 1 is age and sex adjusted, Model 2 is Model 1+surgery and

radiotherapy, Model 3 is Model 2+chemotherapy. Abbreviations: HR – hazard ratio, CI – confidence

interval

**Online Resource Table 2**.

Predictors for missing treatment information

| Variables | Surgery | | Radiotherapy | | Chemotherapy | |
| --- | --- | --- | --- | --- | --- | --- |
|  | OR | 95% CI | OR | 95% CI | OR | 95% CI |
| Age groups |  |  |  |  |  |  |
| 18-49 | Ref. |  | Ref. |  | Ref. |  |
| 50-59 | 0.985 | 0.915- 1.060 | 0.943 | 0.877 - 1.014 | 0.970 | 0.904 – 1.041 |
| 60-69 | 0.978 | 0.914 - 1.047 | 0.980 | 0.917 - 1.047 | 1.038 | 0.973 – 1.108 |
| 70-79 | 1.100 | 1.028 - 1.178 | 1.095 | 1.024 - 1.172 | 1.208 | 1.131 – 1.290 |
| ≥80 | 1.410 | 1.284 - 1.547 | 1.471 | 1.342 - 1.613 | 1.601 | 1.461 – 1.753 |
| Men | Ref. |  | Ref. |  | Ref. |  |
| Women | 1.019 | 0.978 - 1.063 | 1.023 | 0.981 - 1.066 | 0.996 | 0.957 - 1.037 |
| Period 1999-2005 | Ref. |  | Ref. |  | Ref. |  |
| Period 2006-2010 | 1.293 | 1.226 - 1.364 | 1.409 | 1.337 - 1.484 | 1.214 | 1.154 - 1.277 |
| Period 2011-2014 | 1.416 | 1.342 - 1.494 | 1.291 | 1.225 - 1.362 | 1.392 | 1.323 - 1.465 |

The logistic regression model explored predictors for missing treatment information with one model for each treatment modality. Abbreviations: OR – odds ratio, CI – confidence interval, Ref. – reference group
